# Exploring the interactions of rapamycin with target receptors in A549 cancer cells: insights from molecular docking analysis

**DOI:** 10.1007/s00432-024-06072-y

**Published:** 2025-01-07

**Authors:** Sanjeev K. Ganesh, C. Subathra Devi

**Affiliations:** 1 School of Bio Sciences and Technology, Vellore Institute of Technology, Vellore, Tamil Nadu India; 2 Department of Biotechnology, School of Bio Sciences and Technology, Vellore Institute of Technology, Vellore, Tamil Nadu 632 014 India

**Keywords:** Rapamycin, mTORC1, Anticancer, EGFR, Multipotent drug

## Abstract

Rapamycin, a macrocyclic antibiotic derived from the actinomycetes *Streptomyces hygroscopicus*, is a widely used immunosuppressant and anticancer drug. Even though rapamycin is regarded as a multipotent drug acting against a broad array of anomalies and diseases, the mechanism of action of rapamycin and associated pathways have not been studied and reported clearly. Also reports on the binding of rapamycin to cancer cell receptors are limited to the serine/threonine protein kinase mTORC1. Hence, to uncover the exact potential of rapamycin in cancer therapy, a series of cell culture and in silico studies were conducted to identify other receptors capable of binding to rapamycin. Through molecular docking and simulations, it was found that the receptors EGFR, FKBP12, MET, FGFR, ROS1 and ALK were capable of binding with rapamycin. The findings from the current study provides new insights in modern cancer research and therapy. This could also facilitate in understanding the possible action mechanisms of rapamycin in other diseases such as neurovegetative diseases, autoimmune diseases, etc.

## Introduction

Rapamycin is a versatile drug with a 31-membered macrocyclic ring derived from *Streptomyces hygroscopicus* (Zheng and Hong 2019). *Streptomyces rapamycinicus*,* Streptomyces iranensis*,* and Actinoplanes sp. N902-109* are the other strains reported to produce rapamycin (Mark et al., 2018). Rapamycin was first discovered as an antifungal agent against *Candida albicans*, but further research stated the anticancer, antiviral, immunosuppressive and antiaging properties of rapamycin. FDA had approved the use of rapamycin as an immunosuppressant in organ transplanted patients (Patel et al. 2019; Baroja et al., 2016). The unique property of rapamycin to bind with Mammalian target of rapamycin (mTORC1) made it more effective with less side effects in comparison to the calcineurin inhibitors (Montero et al. 2019). mTORC1 regulates many metabolic pathways involved in cell proliferation, genomic synthesis and protein synthesis (Ben-Sahra and Manning, 2017). Abnormalities in mTORC1 leads to the development of cancer, autoimmune disease, diabetes, arthritis etc. (Sorrenti et al. 2022). Previous studies have reported that rapamycin has significantly lesser affinity to mTORC2 as compared to mTORC1 (Afzal et al. 2022).

Deregulation of mTORC1 can collapses the cellular mechanism and can result in uncontrolled cell proliferation and increased rate of cell survival. Rapamycin and its derivatives bind with mTORC1 to control the tumour progression (Ali et al. 2022). Even though all type of cells contains mTORC1, the anticancer activity of rapamycin differ in each type of cells and the reason is still undiscovered. Rapamycin is considered as a miracle drug because of its unique ability to act against different diseases (Hanley et al., 2019). The mechanism of action of rapamycin against several diseases are currently unknown and a lot of research is progressing to study its pharmacological properties and to reveal its modes of action. Recent research on rapamycin showed that rapamycin can inhibit viral proliferation by an unknown mechanism (Ameya and Birri 2023).

Rapamycin is used in combination with other drugs to treat cancer and has found be significantly effective. Previous studies reported that, rapamycin can enhance the anticancer activity of other anticancer drugs. Doxycycline and metformin are the drugs that facilitated higher anticancer activity upon combining with rapamycin (Danko et al., 2021: Zakikhani et al. 2010). Buijsen et al. 2015 reported that a combination treatment of radiotherapy and rapamycin has decreased the metabolic rate of tumour in cancer patients. Rapamycin binds with FKBP12 (FK505 Binding protein) and the resulting complex binds to the mTORC1 through the FRB domain. Rapamycin- FKBP12 loaded complex inhibits the activation of eIF4E/4EBP1 and S6K1 by inhibiting the mTORC1 (Galay, 2017). Downregulation of eIF4E/4EBP1 slows down mRNA transcription and biogenesis. Down-regulation of S6K1 in turn facilitates slower cell growth, cell proliferation and cell survival (Ganesh and Subathra 2023). It disrupts the cell cycle during the G1/S phase, leading to cell cycle arrest in the later stages of G1 and interfering with genome synthesis during the S phase (Hashemolhossein et al., 1998). Rapamycin induces autophagy and apoptosis in cancer cells by the interaction of FKBP12-rapamycin complex with mTORC1 and the mechanism was well reported in the previous research (Zou et al. 2020). The ability of rapamycin to bind to other receptors in the cancer cells and other abnormal cells have not been discovered. Rapamycin has been widely recognized for its role as an mTOR inhibitor, which has led to its use in cancer therapy, particularly for cancers driven by mTOR signalling. However, despite its therapeutic potential, the clinical application of rapamycin faces several limitations. One major challenge is the development of resistance, which occurs when cancer cells acquire mutations or alter signaling pathways to bypass mTOR inhibition (Saxton and Sabatini 2017). As a result, rapamycin’s efficacy in long-term cancer management is compromised. Additionally, rapamycin suffers from poor solubility and bioavailability, often requiring higher doses to achieve therapeutic effects (Ghavami et al. 2014). Unfortunately, higher doses can lead to adverse side effects, including immunosuppression, which limits its safe use in clinical settings (Arriola Apelo & Lamming, 2016). Furthermore, rapamycin’s therapeutic effect is mostly confined to the mTOR pathway, which may not be sufficient in cancers with complex and redundant signaling networks. Many cancers, including lung cancer, involve multiple oncogenic pathways that promote tumor growth, survival, and metastasis (Herbst et al. 2018). This complexity necessitates a broader approach, targeting multiple pathways to improve therapeutic outcomes and overcome resistance (Sato et al. 2020).

Hypothesis of this study is that rapamycin, beyond its established role as an mTOR inhibitor, may interact with other oncogenic receptors involved in cancer progression, thereby expanding its potential therapeutic applications. This hypothesis is driven by the complexity of cancer signaling networks, which often require multi-targeted approaches for effective intervention. Specifically, this study seeks to explore whether rapamycin can exhibit binding affinity to key cancer-related receptors, including EGFR, MET, FGFR, ROS1, and ALK, and to characterize the molecular interactions underlying these potential bindings.

## Materials and methods

### Microbial strains

In the current study, *Streptomyces hygroscopicus* (MTCC 1105) strain was revived in a medium composed of the following (per Litre): beef extract (12 g), peptone (2 g), yeast extract (2 g), tryptose (2 g), CaCo_3_ (100 mg), starch (100 mg); glucose: (10 g), trace elements CoCl_2_ and ferric ammonium citrate. (Dutta et al., 2014). The medium was seeded with a lyophilized culture of *S. hygroscopicus* and incubated at 28 °C for a period of 7 days, at a pH of 7.2 throughout the incubation period.

### Rapamycin production from *S. hygroscopicus*

Sugarcane juice (150mL/L), soya powder (20 g/L) and tomato (20 g/L) were used to prepare the production medium. *S. hygroscopicus* (2%) was inoculated and incubated for a period of 7 days and at 28 °C. The media was centrifuged at 8000 rpm for 6 min and collected the cell free supernatant. Rapamycin production was assessed using the well-diffusion method against *Candida albicans* (Ganesh and C 2023).

### Extraction of rapamycin

After incubation, biomass of *S. hygroscopicus* was ruptured using ultrasonication and removed by centrifugation at 8000 rpm for 6 min followed by filtration using Whatman filter paper and the supernatant was collected. Rapamycin was extracted from cell free supernatant using toluene extraction method. Oily residue with rapamycin was dissolved in 50 mL of HPLC grade methanol. The aqueous solution of rapamycin was concentrated using rotary evaporator and dried to powder form (Rani et al. 2013).

### Quantification of rapamycin using ultra-performance liquid chromatography (UPLC)

UNISON UK C18 column of 3 μm diameter and 4.6 mm × 250 mm length was used for the chromatographic separation. The analysis was conducted while maintaining a flow rate of 1.0 mL/min and utilizing a wavelength of 278 nm. The mobile phase solvent composition consisted of 80% solvent A and 20% solvent B (HPLC-grade water). Solvent A consisted of 80% HPLC-grade methanol and 20% HPLC-grade acetonitrile.Twenty minutes run time was set to determine the rapamycin content in the extract. Rapamycin in the extracted sample was confirmed by comparing the standard rapamycin peak (Rani et al. 2013).

### Cell culture

The cell lines L929, A549, Hela, MCF7, and MG63 were obtained from the National Centre for Cell Sciences (NCCS), Pune, India and cultured in Dulbecco’s modified Eagles medium, DMEM (Sigmaaldrich, USA). The cell lines were cultured and sustained in DMEM, which was supplemented with 10% FBS, L-glutamine, sodium bicarbonate (obtained from Merck, Germany), and an antibiotic solution comprising penicillin (100 µg/mL), streptomycin (100 µg/mL), and amphotericin B (2.5 µg/mL). Cells were incubated at 37ºC in a humidified 5% CO_2_ incubator (Yoonus et al. 2021). Twenty-four h old, 100µL cell suspensions (5 × 10^3^ cells/well) were suspended in 500µL growth medium in 96 well tissue culture plate. Five different concentrations of rapamycin in µg/mL (100, 50, 25, 12.5, 6.25) was prepared in DMSO and suspended in each well and then incubated at 37ºC in a humidified 5% CO_2_ incubator. All treatments were carried out in triplicate, and non-treated control cells were also maintained alongside. (Yoonus et al. 2021).

### Anticancer screening via direct microscopic observation

After 24 h of treatment, plates were examined using an inverted phase-contrast tissue culture microscope (Olympus CKX41 equipped with Optika Pro5 CCD camera). Any alterations in cell morphology, such as cell rounding or shrinkage, cytoplasmic granulation, and vacuolization, were documented. (Beevi et al. 2010).

### Anticancer assay by MTT method

Cytotoxic effect of rapamycin against a normal cell line (L929) and four different cancer cell lines, A549, Hela, MCF7 and MG63 was determined using MTT assay ((3-[4,5-dimethylthiazol-2-yl]-2,5 diphenyl tetrazolium bromide). MTT (15 mg) was reconstituted in 3 mL PBS until completely dissolved and filtered using syringe filter.

After 24-hours incubation period, the samples of the wells were aspirated, and 30µL of reconstituted MTT solution was added to all test and control wells. Subsequently, the plates were gently shaken and incubated at 37ºC in a humidified 5% CO2 incubator for 4 h. The supernatant was discarded after the incubation and 100µL of MTT solubilization solution (Dimethyl sulphoxide, DMSO, Sigma Aldrich, USA) was added. Absorbance values were then measured using a microplate reader at a wavelength of 540 nm. The percentage of growth inhibition was determined using a formula, and the lethal concentration 50 value (LC50) was calculated utilizing ED50 PLUS V1.0 Software. All experiments were conducted in triplicate, and the results were expressed as Mean+/- SE. Statistical analysis was performed using one-way ANOVA and Dunnets test, with ***p < 0.001 indicating significant difference compared to control groups (Bilal et al. 2021).

% viability = $$\:\frac{Mean\:OD\:samples\:\times\:100}{Mean\:OD\:of\:control\:group}$$

### Molecular docking

ALK, EGFR, FGFR, MET, and ROS1 receptors were selected based on their known roles in cancer progression and therapeutic relevance, particularly in lung cancer. These receptors are commonly overexpressed or mutated in various cancers, where they play key roles in promoting cell proliferation, survival, metastasis, and angiogenesis. These receptors are often implicated in resistance to single-pathway inhibitors, such as mTOR inhibitors, due to their ability to activate alternate signaling pathways, which can sustain tumor growth even in the presence of targeted therapies. Given rapamycin’s established role as an mTOR inhibitor, our study seeks to explore whether it may also interact with these receptors, potentially providing additional therapeutic effects through multi-target binding.

The binding affinity of rapamycin on A549 cancer cell receptors such as ALK (PDB Id: 4JOA), EGFR (PDB Id: 1M14), FGFR (PDB Id: 4QQC), MET (PDB Id: 5UAB) and ROS1 (PDB Id: 5FTO) were determined by using molecular docking. The binding score of rapamycin with each receptor were compared with the FDA approved specific drugs against lung cancer. The structure of positive control drugs (ligands) and rapamycin (ligands) was downloaded from pubchem database. Ligands for each receptor were (i)ALK: alectinib, ceritinib and crizotinib (ii) EFGR: erlotinib, gefitinib and osimertinib (iii) FGFR: dovitinib, lucitanib and ponatinib (iv) MET: crizotinib, foretinib and selumetinib (v) ROS1: crizotinib, entrectinib and lorlatinib. Rapamycin was docked with FKBP12 complex as a positive control to compare the binding affinities of rapamycin with other receptors. Discovery Studio software (V 21.0.20298) was used to remove the water molecules and already bound ligands from the receptors. AutoDockTools-1.5.7 was used to repair the missing atoms and to add Kollman charges and polar hydrogens. The prepared receptor PDB file was converted to PDBQT file and saved in the specific folder (Thomas et al. 2023). Binding sites were determined using CASTp 3.0 analysis (Binkowski et al., 2023). Grid box was adjusted to estimate the center x, y and z and dimension x, y and z. Canonical smile format of ligands were extracted from pubchem. An Online SMILES Translator and Structure File Generator were utilized to generate the PDB format of the ligand. PDB format of the ligands were converted to PDBQT by using AutoDockTools-1.5.7 and saved in the specific file. Vina was used to determine the docking affinity of ligands with receptors. Vina split was used to split the values in the generated output file (Wrobel et al. 2021). All the receptor-ligand binding was visualized in the Discovery Studio software (V 21.0.20298) (Table 1).

**Table 1:** Molecular docking parameters for rapamycin with each receptor, detailing binding site coordinates and grid dimensions

Si. No	Receptors	Centre X	Centre Y	Centre Z	Size X	Size Y	Size Z
1	ALK	12.52	16.195	45.558	82	62	70
2	EGFR	29.33	13.361	58.824	86	108	104
3	FGFR	-21.066	5.515	-2.26	80	62	56
5	MET	-10.313	6.982	19.549	72	60	19.549
6	ROS1	12.08	13.123	9.394	78	58	66
7	FKBP12	9.552	1.9	13.985	58	52	54

### Root-mean-square fluctuation (RMSF) simulation to analyse the stability of the complex

The CABSflex interface (http://biocomp.chem.uw.edu.pl/CABSflex2) was employed to compute the residue-level fluctuations of rapamycin in association with ALK, EGFR, FGFR, MET, and ROS1 receptors. The analysis of residue-level propensity utilizing Root Mean Square Fluctuation (RMSF) trajectories was performed with default restraint parameters. A 10-nanosecond timescale was selected to evaluate the consensus protein variations in an aqueous environment. The minimum conformational distance of 3.8 and maximum conformational distance of 8.0 and a gap of 3 were selected (Ameji et al. 2023; Swetha et al. 2022).

### Statistical analysis

One-way ANOVA was used to assess differences between groups, followed by post-hoc testing using Tukey’s HSD test to determine pairwise comparisons. A significance level of *p* < 0.05 was set to identify statistically significant differences. All statistical analyses were conducted using GraphPad Prism, which ensured accurate and reproducible results.

## Results and discussion

### Quantification of rapamycin production

**Fig. 1:**
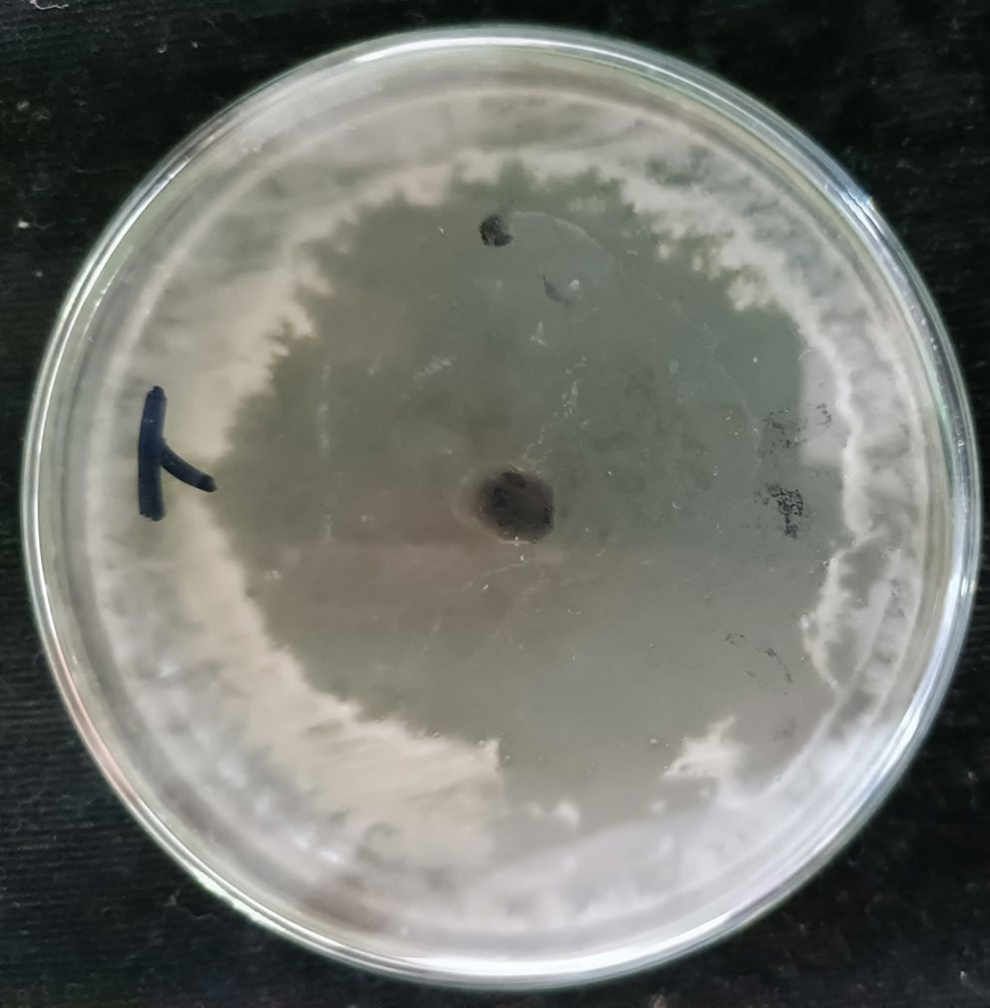
Antimicrobial activity of rapamycin against *C. albicans*

Cell free supernatant of *S. hygroscopicus* showed a zone of inhibition of 45.2 ± 1.3 mm against *C. albicans.* Confirmation of rapamycin production was evidenced by the zone of inhibition against *C. albicans*. Comparative analysis between previous investigations and the current study revealed a consistent and equivalent zone of inhibition against *C. albicans*. The similarity observed in the zone of inhibition between the current study and the referenced literature further strengthens the statement that the inhibitory effects are indeed attributed to rapamycin. Figure 1 represents the zone of inhibition against *C. albicans* (Rosenberg et al. 2018).

### Extraction and determination of rapamycin

After toluene extracted rapamycin was concentration and subjected to UPLC. The extracted rapamycin showed a peak at retention time 6.189 min similar to the peak observed in the standard rapamycin. Thus, the rapamycin content in the extract was confirmed. In the previous studies, the use of High-Performance Liquid Chromatography (HPLC) unveiled a retention time of 17.026 min for rapamycin. In a recent article, a transition to UPLC was described, unveiling a distinct rapamycin peak observed at a retention time of 6.3 min. Remarkably, this value is very much close to the retention time observed in the UPLC analysis of rapamycin (Rani et al. 2013; More et al. 2017).

### Anticancer assay by direct microscopic observation

**Fig. 2:**
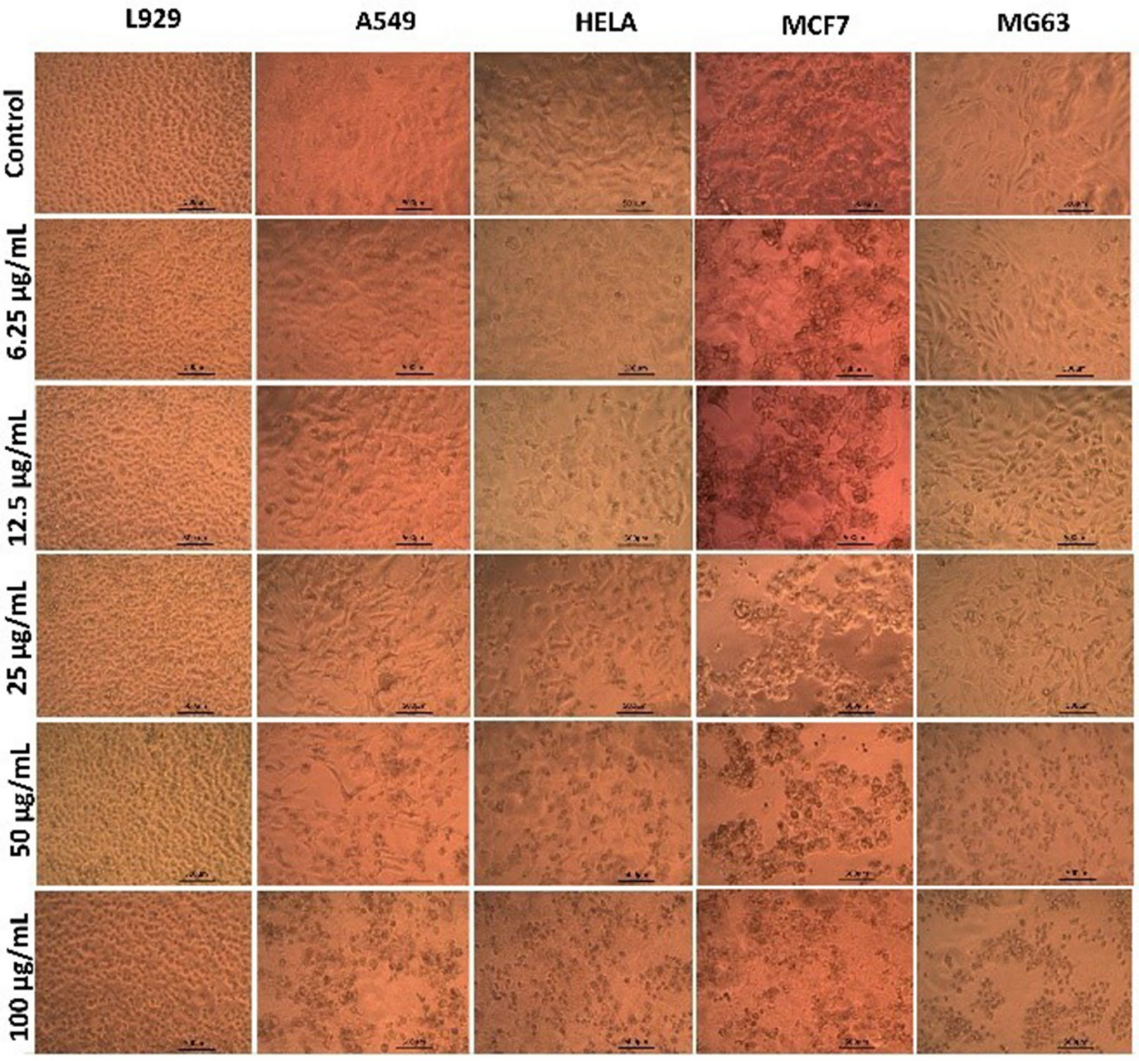
Morphological changes in cancer cell lines (A549, HeLa, MCF7, and MG63) after 24-hour rapamycin treatment. Cancer cells exhibit reduced elongation and spreading, transforming to a round, shrunken morphology indicative of apoptosis. No significant changes were observed in the normal cell line (L929) after treatment. (scale bar = 100 μm)

The phenotypic characteristics of rapamycin treated cancer cell lines (A549, Hela, MCF7 and MG63) were analysed using inverted phase contrast tissue culture microscope. After 24 h of treatment with rapamycin, significant morphological alterations were observed in the cancer cell line. This indicates the cell death and growth inhibition. No significant changes were observed in the normal cell line (control L929 cells) after 24 h treatment with rapamycin. Figure 2 represents the changes in morphology of cancer cell lines and normal cell line upon treatment with different rapamycin concentrations. Rapamycin treatment significantly reduced the cell elongation and spreading in the cancer cell lines. After treatment, the cancer cell lines underwent significant changes in its morphology. The hexahedron cells were found to have change to round and shrunken morphology. Additionally, the rapamycin treatment induced apoptosis which prevented cell divide.

Detachment of cancer cell lines from the culture plates were also observed after treatment with rapamycin. Beevi et al. in 2010, reported that detachment of cancer cell lines from the culture plate is a common characteristics of cancer cell lines that has undergone apoptosis. In the present study, rapamycin had significant effect on the cancer cell lines which was evident from the morphological alterations (Beevi et al. 2010).

### Anticancer assay by MTT method

Cytotoxic effect of rapamycin was studied a_g_ainst four different cancer cell lines and the most significant effect was observed against A549 lung cancer cell line. Rapamycin showed a potent impact with LC_50_ value of 32.99 ± 0.10. In Hela cancer cell line also rapamycin showed significant cytotoxicity, however it was lower than that on A549 cell line. Findings from a recent study by Wang et al. (Wang et al. 2021) aligns with these observations and thus supports the current data. It reported that rapamycin induced cytotoxicity in the A549 cancer cell lines, by inducing G0/G1 phase arrest in the cell cycle. In Hela cell line, LC_50_ value of 37.34 ± 14 was observed, which is a significant score against the treatment of cervical cancer (Hela). A moderate cytotoxicity response was observed in the osteoblast cell line MG63 with an LC_50_ value of 48.84 ± 10. MCF 7 breast cancer cell line showed comparatively lower cytotoxicity among the five tested cancer cell lines. A LC_50_ value of 66.72 ± 50 was observed in MCF-7 cancer cell line. The control cell line L929 showed LC_50_ value of 100.93 ± 10. The comparative study of rapamycin’s cytotoxic effect against various cancer cell lines demonstrate that rapamycin have significant effectiveness in treating lung cancer.

**Fig. 3:**
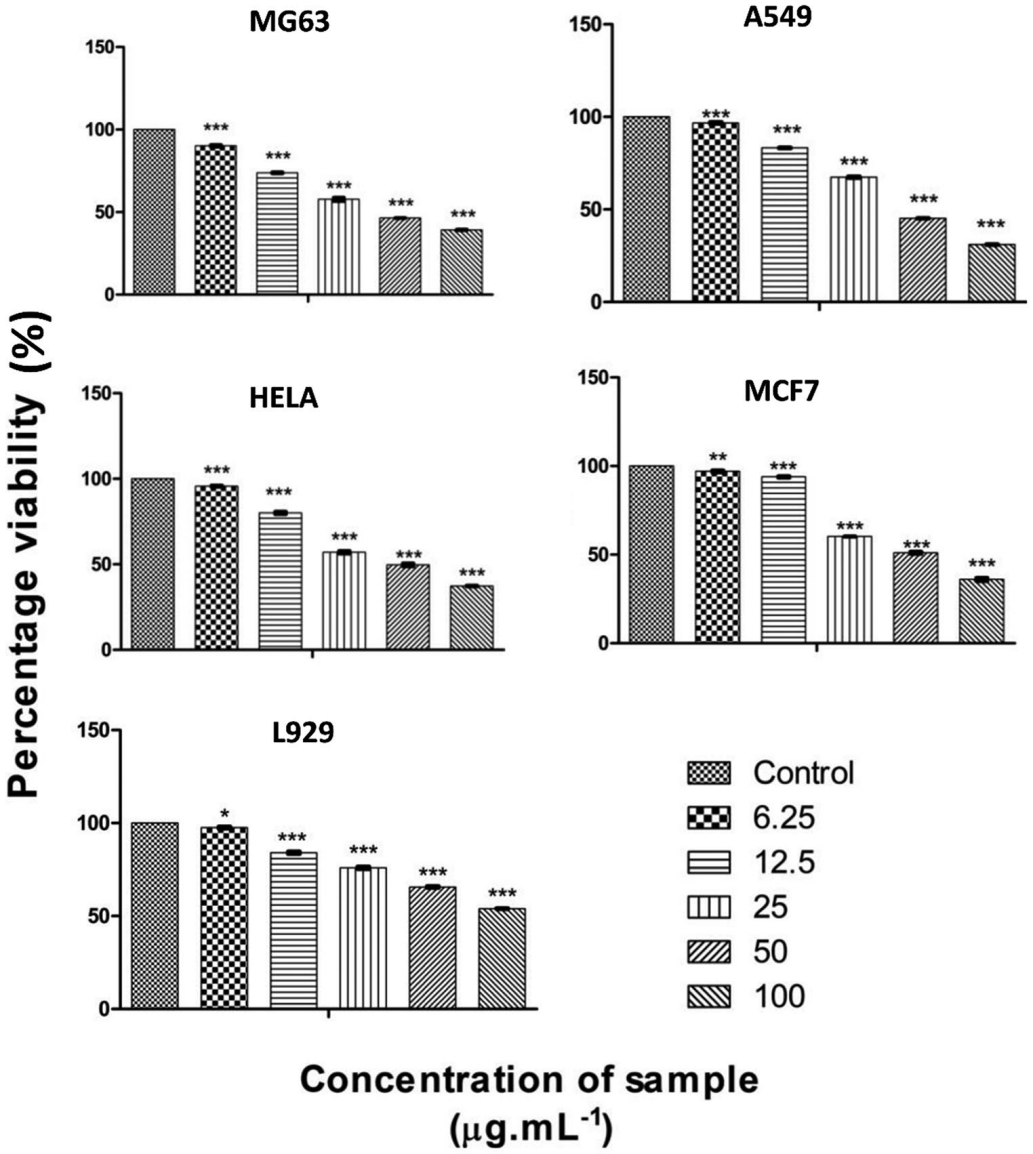
Cytotoxic effect of rapamycin against different cancer cell lines

The lower LC_50_ value of 32.99 ± 0.10 against A549 indicated a high degree of susceptibility to rapamycin-induced cytotoxicity, when compared to the other cancer cell lines. The lower cytotoxicity against L929 normal cell line underscores the potential selectivity of rapamycin for cancer treatment. Previous reports stated that interaction of rapamycin with mTOR affects the cell cycle and resulted in the cell cycle disturbance. This disturbance in the cell cycle leads to an increased cell toxicity in the cancer cell line. Rapamycin induced cytotoxic effect by promoting apoptosis and autophagy. Woo et al. 2021 reported that rapamycin elevated the amount of ROS in the tumour cells and increased the rate of cell death. Recent studies reported that rapamycin induced cell death is predominantly due to autophagy than apoptosis. Hence the intercellular organelles and molecules were recycled to maintain the cellular homeostasis (Wang et al.,^2022^: Fan et al.,^2013^). Xie et al. 2013 reported that p62 and LC3II proteins that mediate autophagy were observed in the cancer cell lines treated with rapamycin. The relation observed between cytotoxicity in the current study and the known anticancer effects of rapamycin proved that the impact on cancer cell lines were particularly due to the administered drugs. The current study aimed to demonstrate the potential variability of rapamycin treatment effects among different cancer receptors (Fig. 3).

### Molecular docking analysis

The current study analysed the affinity of rapamycin to bind with various receptors beyond its known inhibition mTOR complex 1. Molecular docking analysis was conducted on ALK, EGFR, FGFR, MET, and ROS1 receptors in the cancer cells. The binding affinity of rapamycin to EGFR receptors (-8.8 Kcal/mol) was greater than the binding affinity of EGFR positive controls, including gefitinib (-6.3 Kcal/mol), osimertinib (-6.1 Kcal/mol), and erlotinib (-4.9 Kcal/mol). These observations suggest that, its strong affinity to EGFR can enhance the effectiveness to supress the cancer with other EGFR targeting anti-cancer drug. Affinity of rapamycin with MET receptor was analysed and observed that rapamycin exhibited a docking affinity of -8.3 Kcal/mol. The positive control crizotinib (-8.6 Kcal/mol) exhibited a higher docking affinity. Foretinib (-8.1 Kcal/mol) and selumetinib (-7.1 Kcal/mol) showed lesser affinity to the MET receptor than the rapamycin. Rapamycin showed a binding affinity of -8.1 Kcal/mol for FGFR receptor and rapamycin exhibited a substantial binding in comparison with the positive controls dovitinib (-8.1 Kcal/mol), lucitanib (-8.6 Kcal/mol) and ponatinib (-7.7 Kcal/mol). The affinity of rapamycin with ROS1 was found to be -7.7 Kcal/mol which indicated that rapamycin is a potent inhibitor of ROS1 inhibitor like positive controls crizotininb (-8.5 Kcal/mol), entrectinib (-9.1 Kcal/mol) and lorlatinib (-9.0 Kcal/mol). Among the five receptors, rapamycin showed least affinity on ALK receptor (-7.5 Kcal/mol). Positive controls, alectinib (-8.6 Kcal/mol), ceritinib (-8.5 Kcal/mol) and crizotinib (-7.8 Kcal/mol) indicated much higher affinity with the ALK receptor. Docking score of rapamycin indicated its potential to bind with ALK receptor. Also, along with the control FKBP12, rapamycin demonstrated similar binding affinities with all the tested receptors. A higher binding affinity for EGFR (-8.8 Kcal/mol) was observed with respect to the control FKBP12 (-8.2 Kcal/mol). The findings of this study suggest that rapamycin may possess multifaceted inhibitory effects on various receptors beyond mTORC1, showcasing potential applications in inhibiting EGFR, MET, FGFR, ROS1, and ALK-mediated pathways in lung cancer cells. Previous studies reported that, expression of certain receptors in cancer cells will be higher than the normal cells. Expression of ALK, EGFR and ROS1 are highly expressed in in lung cancer. Higher affinity of rapamycin with these receptors might the reason for higher cytotoxicity of rapamycin on the lung cancer cell lines. ALK, EGFR, FGFR, MET, and ROS1 had a significant role in suppressing the tumor (Desai et al. 2016). FKBP 12 receptor of mTORC1 was considered as the only domain for rapamycin binding site (Panwar, 2023). No other studies have reported other active sites of rapamycin at present. The current study highlights that, rapamycin have significant binding affinity towards other unexplored receptors. Gefitinib, crizotininb, dovitinib, ceritinib etc. are the drugs used against the receptors to supress the cancer (Desai et al. 2016). Formulation of new combination of these anti-cancer drugs with rapamycin can enhance the anti-cancer activity. Rapamycin can act on multiple receptors on the cancer cells and induce enhanced antitumor activity. Previous studies reported that, combination of rapamycin with metformin, suberoylanilide hydroxamic acid and docetaxel had significant impact on anticancer effect (Wang et al. 2021; Zang et al., 2018: Niu et al. 2011).

Rapamycin is widely recognized for its selective inhibition of mTORC1, with steric hindrance limiting its ability to interact effectively with mTORC2 (Zhou et al. 2019). However, its macrocyclic lactone structure confers a degree of binding flexibility that can enable interactions with additional non-mTOR targets under specific conditions (Schreiber et al. 2021). In this study, we identified strong binding affinities of rapamycin with ALK, EGFR, MET, FGFR, and ROS1, receptors commonly overexpressed in lung cancer cells. Prior studies have demonstrated that macrolide compounds like rapamycin can adapt to various binding sites, expanding their potential target profiles beyond mTOR (Wang et al. 2020; Sun et al. 2022). This flexibility suggests that, despite its structural size, rapamycin could effectively engage with these receptors, providing therapeutic benefits in cancers with complex receptor landscapes, such as lung cancer. Further in vivo studies are essential to validate these non-canonical interactions and to elucidate their therapeutic impact in the context of lung cancer treatment.

**Fig. 4:**
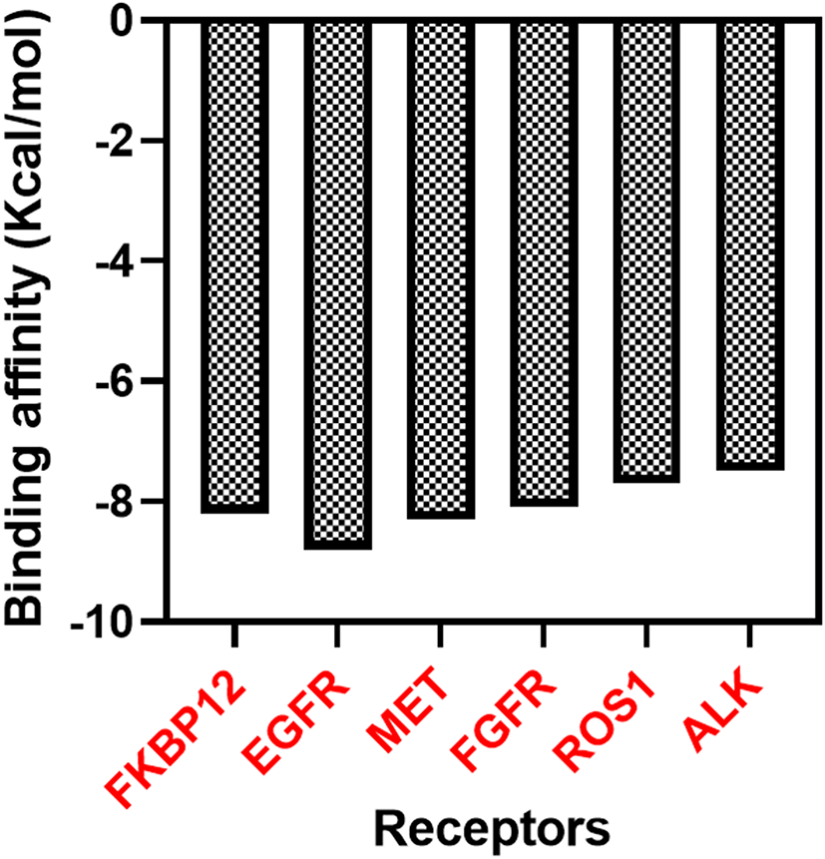
Binding affinity of rapamycin with different receptors

**Fig. 5:**
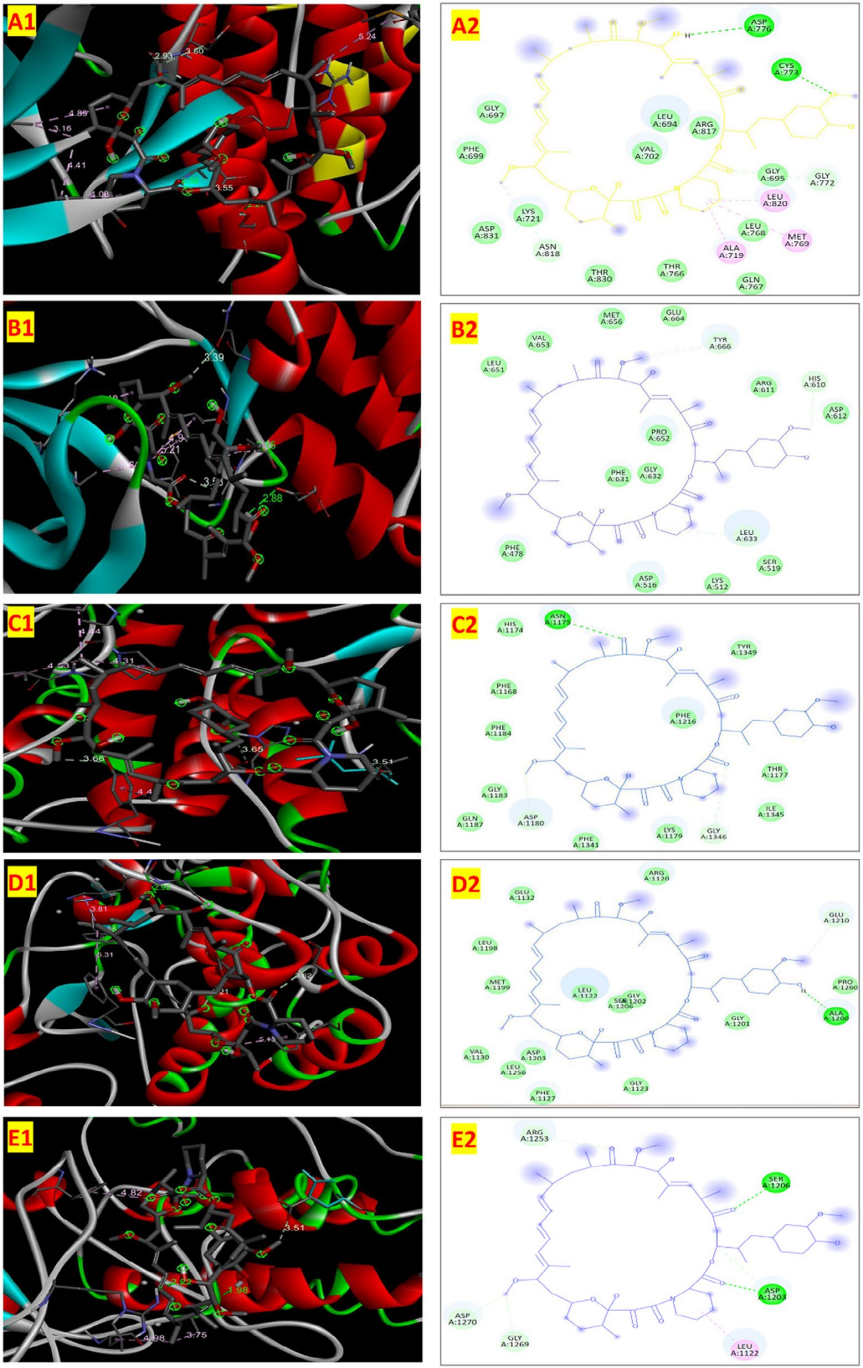
2D and 3D molecular docking interactions of rapamycin with oncogenic receptors: (**A**) ALK, (**B**) EGFR, (**C**) FGFR, (**D**) MET, and (**E**) ROS1. The 3D images show rapamycin’s positioning within the receptor binding pockets, highlighting key hydrophobic and hydrogen bond interactions. The 2D images provide details on specific bonds and interacting residues. Rapamycin exhibits stable binding with ALK, EGFR, and MET, while showing less stable interactions with FGFR and ROS1, supporting its variable efficacy across these cancer-related pathways

Rapamycin’s binding with multiple receptors (ALK, EGFR, FGFR, MET, and ROS1) may extend its therapeutic benefits by targeting various oncogenic pathways; however, this also raises concerns about potential off-target effects on non-cancerous cells that express these receptors. While the in-silico analysis in the current study supports rapamycin’s stable binding. Further validation in non-cancerous cell models and animal studies is necessary to assess safety and mitigate unintended interactions. To address these challenges, future studies could explore receptor-specific analogs or targeted delivery systems, aiming to optimize rapamycin’s efficacy while minimizing risks to normal tissues (Figs. 4, 5).

#### Root mean square fluctuations simulation

The Root Mean Square Fluctuation (RMSF) analysis provided valuable insights into the stability of the rapamycin-receptor complexes. In this study, rapamycin showed lower RMSF values with ALK, EGFR, and MET receptors compared to the positive controls, indicating reduced fluctuations in these complexes. This lower fluctuation suggests a more stable binding configuration, potentially translating to stronger, more sustained interactions in a physiological context. According to Swetha et al. (2022), compounds with lower RMSF values and binding affinities are often more stable, which aligns with our observations for rapamycin with these receptors. The stability observed here indicates that rapamycin might maintain its binding conformation effectively with ALK, EGFR, and MET, enhancing its potential efficacy through reliable receptor interaction.

**Fig. 6:**
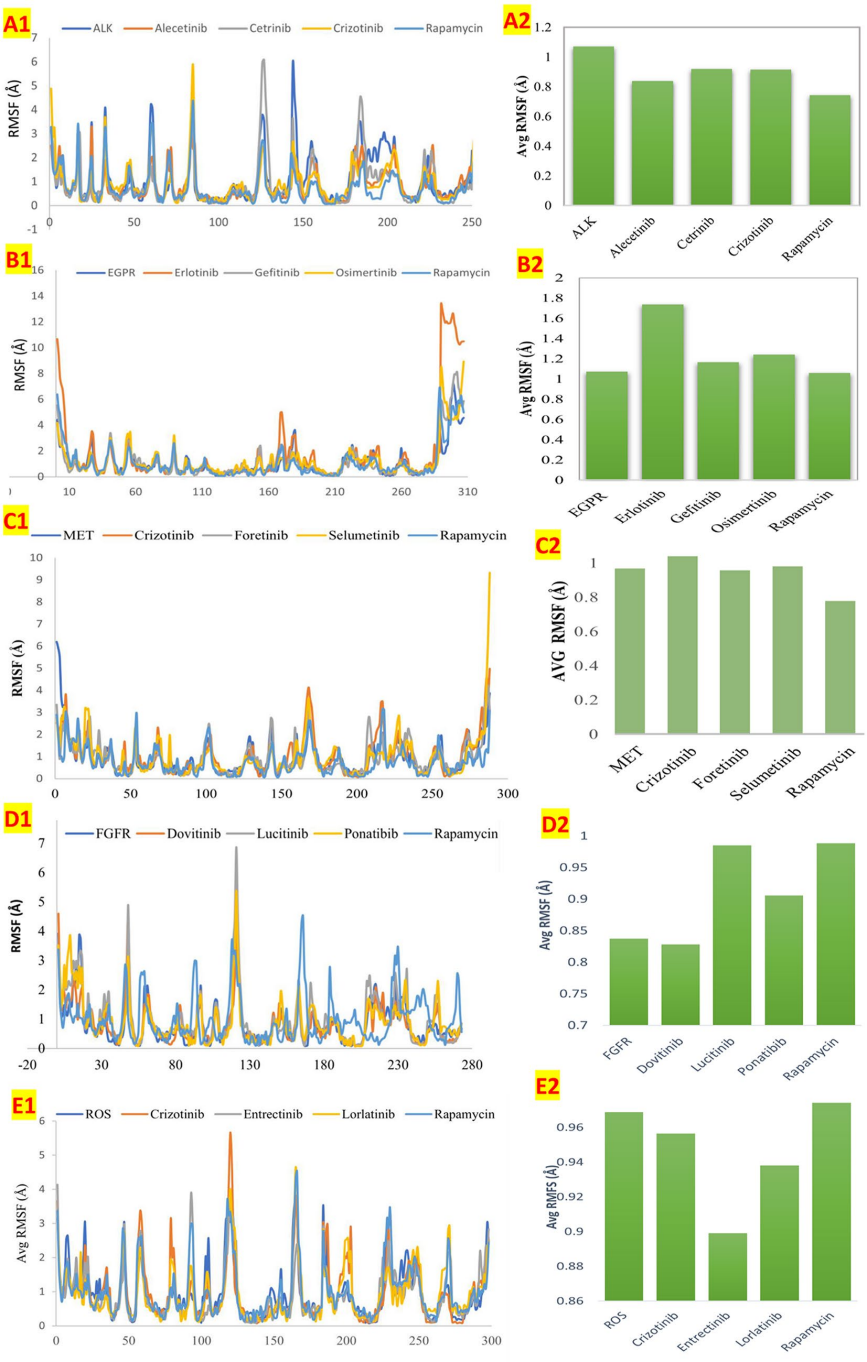
RMSF analysis of rapamycin-receptor complexes. The graph shows RMSF values of rapamycin with ALK, EGFR, MET, ROS1, and FGFR receptors compared to controls. Lower RMSF values with ALK, EGFR, and MET suggest stable binding, while higher values with ROS1 and FGFR imply less stable interactions. This indicates variable binding stability of rapamycin across different receptors

In contrast, higher RMSF values with ROS1 and FGFR receptors suggest increased fluctuation, which may imply weaker or less stable binding under physiological conditions. This difference may affect the binding persistence of rapamycin with ROS1 and FGFR, potentially reducing its efficacy with these receptors compared to ALK, EGFR, and MET. The RMSF simulation graph in Fig. 6 provides a visual comparison of these fluctuations, highlighting rapamycin’s stability across different receptor complexes relative to the positive controls.

## Conclusion

Rapamycin is an mTORC1 inhibitor which affect the cell proliferation, mRNA transcription and protein synthesis. In this study, it was observed that rapamycin showed different level of cytotoxicity towards different cancer cell lines. Among the other cancer cell lines, rapamycin showed higher cytotoxicity to lung cancer cell line. In-silico analysis was performed to identify the reason behind the higher cytotoxicity of rapamycin on lung cancer (A549 cancer cell line). For the first time, the current study reported that rapamycin has a binding affinity with ALK, EGFR, FGFR, MET, and ROS1 receptors expressed in the lung cancer cells. Using RMSF simulation analysis, rapamycin was found to be more stable in ALK, EGFR and MET than other receptors. Binding of rapamycin with these over expressed receptors in the lung cancer will increase the cytotoxic effect in the A549 lung cancer cell line. In the current study, it was found that rapamycin can bind with receptors other than mTORC1 and carry out different functions.

Rapamycin is a versatile drug against different disorders like cancer and neurodegenerative abnormality. However, the mechanism by which rapamycin maintains the haemostasis in cancer and neurodegenerative disease is unclear. The current study states that rapamycin can bind with multiple receptors with in the same cell. Given rapamycin’s versatility in treating disorders like cancer and neurodegenerative diseases, future research should focus on in vivo studies to confirm these interactions within complex physiological environments. Such studies will be crucial to further elucidate rapamycin’s multi-receptor mechanism and its role in maintaining cellular homeostasis across disease contexts.

While molecular docking and RMSF analyses offer valuable initial insights into rapamycin-receptor interactions, these findings are predictive and do not fully replicate the complexities of in vivo environments. We emphasize the need for further experimental validation, such as in vitro binding assays and in vivo studies, to confirm the stability and therapeutic relevance of these interactions. This addition acknowledges the potential limitations of computational methods and underscores the importance of complementary validation.

## Data Availability

No datasets were generated or analysed during the current study.
